# Stramonium in Dysmenorrhœa

**Published:** 1856-03

**Authors:** 


### "STRAMONIUM IN DYSMENORRHŒA."

We would desire to call the particular attention of our readers to the article in this number of the Journal, from the pen of C. W. Ashby, M. D., of Alexandria, Va., one of the most eminent and practical medical men of that State, and favorably known among the highest medical circles in the United States. We can also bear some testimony to the value of Stramonium, (from a limited experience with it,) under the circumstances indicated, and believe that we first derived the suggestion, which induced us to use it, from the author of this article, who, though claiming no originality, is entitled to great credit for bringing to the notice of the profession, with such discriminating judgment, an old, though as we are certain, a non-appreciated agent of great value, in the treatment of what he very appropriately, terms "Membranous Menstruation," one of the most distressing, frequent, and intractable maladies to which the female, is subject.

In reference to the use of Guaicum, it is a remarkable medical fact, as suggested by our correspondent, that the experience of the profession, with that article, has not at all sustained the reputation which was given to it, by the praises of the distinguished Dewees; and we would suggest, (which is probably not original with us,) that it is only in that form of difficult, and painful menstruation, connected with Rheumatic disease, that the Guaicum is at all beneficial; in which condition it is doubtless a valuable remedy.

